# Factors Influencing the Uptake of Agroforestry Practices among Rural Households: Empirical Evidence from the KwaZulu-Natal Province, South Africa

**DOI:** 10.3390/f14102056

**Published:** 2023-10-14

**Authors:** Fortunate Nosisa Zaca, Mjabuliseni Simon Cloapas Ngidi, Unity Chipfupa, Temitope Oluwaseun Ojo, Lavhelesani Rodney Managa

**Affiliations:** 1 African Centre for Food Security, School of Agricultural, Earth and Environmental Sciences, College of Agriculture, Engineering and Science, University of KwaZulu-Natal, Private Bag X01, Scottsville, Pietermaritzburg 3201, South Africa; 2 Centre for Transformative Agricultural and Food Systems, School of Agricultural, Earth and Environmental Sciences, College of Agriculture, Engineering and Science, University of KwaZulu-Natal, Private Bag X01, Scottsville, Pietermaritzburg 3201, South Africa; 3 Department of Agricultural Extension and Rural Resource Management, School of Agricultural, Earth and Environmental Sciences, College of Agriculture, Engineering and Science, University of KwaZulu-Natal, Private Bag X01, Scottsville, Pietermaritzburg 3201, South Africa; 4 Department of Agriculture and Animal Health, School of Agriculture and Life Sciences, University of South Africa, 28 Pioneer Ave, Florida Park, Roodepoort 1709, South Africa; 5 Department of Agricultural Economics, Obafemi Awolowo University, Ile-Ife 220101, Nigeria; 6 Disaster Management Training and Education Centre for Africa, University of the Free State, Bloemfontein 9301, South Africa; 7 Human Sciences Research Council, Africa Institute of South Africa, 134 Pretorius Street, Pretoria 0002, South Africa

**Keywords:** agroforestry practices, climate-smart agriculture, climate change, adoption, theory of planned behaviour, rural households

## Abstract

Agroforestry is recognized as a significant element in climate-smart agriculture due to its high potential for addressing food insecurity, climate change challenges, and ecosystem management. However, despite the potential benefits of agroforestry practices, the adoption by rural households in Sub-Saharan Africa is low. Adopting agroforestry practices requires understanding rural households’ socio-economic and socio-psychological factors. Hence, this study empirically examined the role of knowledge, attitudes, and perceptions in the uptake of agroforestry practices among rural households to better understand the adoption process. A sample of 305 households was obtained from three communities, namely, Swayimane, Umbumbulu, and Richmond, in KwaZulu-Natal province. Principal component analysis and a binary logistic regression model were employed to analyze the data. Knowledge, attitudes, and perceptions towards agroforestry were found to positively influence the adoption of agroforestry practices. The results also revealed that age, farming experience, education level, and land size were determinants of agroforestry adoption. Therefore, the study recommends that policymakers, extension officers, and climate change champions consider rural households’ socio-economic characteristics, knowledge, attitudes, and perceptions when designing agroforestry projects. Implementing training programs with practical demonstration is also recommended to increase awareness of the benefits of agroforestry practices and encourage rural households to protect on-farm trees and shrubs.

## 1 Introduction

One of the key environmental challenges faced by the modern world currently is that climate change and its effects are rapidly mounting [1,2]. Future predictions indicate that changing climate will result in lower rainfall and higher temperatures with increased flooding and drought events in South Africa [3]. Most studies report that the source of livelihood affected mainly by climate change is agriculture, especially crop productivity [4–6]. Agricultural production activities in Africa (South Africa included) are more vulnerable to climate change than other production constraints [7,8]. This adversely affects rural households who largely depend on farming. For example, the number of hungry people globally is expected to rise by 20% by 2050 due to the adverse impacts of climate change on agricultural production and the lives of rural households [9].

According to [10], the agricultural sector is among the most substantial contributors to changing climate. Globally, the sector contributes approximately 20% to greenhouse gas emissions directly through agricultural practices and indirectly via land use alteration [11,12]. With rising food demand resulting in the need for increased food production, agriculture is projected to be a primary source of emissions growth, which threatens future food security [13,14]. The impact of changing climate on the agricultural sector, combined with the impact of agriculture on greenhouse gas emissions necessitates adaptation strategies that will lessen the negative impact of agricultural production while mitigating climate change [10,12]. Climate-smart agriculture (CSA) is recognized as the most suitable adaptation strategy to accomplish these objectives. It is defined as a transformative and sustainable agricultural strategy that aims to jointly address food insecurity, climate variability challenges, and ecosystem management [15–17].

The CSA practices include cultivation of cover crops, rotational cropping, agroforestry, conservation agriculture, crop diversification, use of organic manure, planting drought and heat-resistant crops, small-scale irrigation farming, and mulching [18,19]. Agroforestry is one of the few land use strategies with the capacity to deliver all three benefits of CSA [20,21]. It is defined as a farming practice that integrates trees and shrubs with agricultural crops and/or livestock, or both [22,23]. Moreover, it is recognized as a significant element in CSA due to its high potential for building resilience to climate change, carbon sequestration, and strengthening rural livelihoods [24,25]. Resilience to changing climate is improved through increased tree cover, carbon sequestration, agricultural productivity, and household income [25]. According to [26], agroforestry practices are categorized into *agrisilvicultural* (trees/shrubs and crops), *silvopastoral* (trees/shrubs and livestock), and *agrosilvopastoral* (trees/shrubs, crops and livestock). Therefore, households with trees/shrubs around their farmland (e.g., windbreaks and fences), combinations of trees, crops, and livestock around homesteads, and who used trees/shrubs as shelterbelts for livestock were considered to be involved in agroforestry practices in this study [27].

Regardless of the potential benefits of agroforestry practices, adoption by small-scale farmers in Sub-Saharan Africa is low [23,28]. Following [22], in this study, adoption is defined as a decision to make full use of an agroforestry technology. Low adoption of agroforestry programs is due to minimal emphasis placed on understanding local communities’ knowledge, attitudes, and perceptions. Although knowledge, attitudes, and perceptions studies on the adoption of innovations have been carried out since the 1980s [28], there is a lack of such studies focusing on the decision-making process of agroforestry adoption in rural areas, particularly in South Africa. This, in turn, presents challenges regarding planning, investments, and formulation of relevant policies that can enhance resilience to changing climate. One possible reason for the lack of research in this field may be the methodological challenges associated with measuring individual’s perceptions of agricultural practices [28].

The analysis conducted by [28] emphasized that both extrinsic variables (e.g., characteristics of the adopter, characteristics of the innovation, and the external environment) and intrinsic variables (e.g., knowledge, perceptions, and attitudes) influence the decision to adopt new agroforestry technologies. The theoretical literature also justifies that both variables have a key influence on rural households’ decision to adopt agroforestry practices. For example, an individual’s characteristics and economic variables may influence adoption indirectly by affecting the knowledge, attitudes, and perceptions, which in turn influence the decision to adopt an innovation. Moreover, socio-economic and demographic factors such as the household head’s age, education level, farming experience, employment status, and access to agricultural extension services are drivers of individuals’ decisions to adopt agroforestry in most resource-poor communities [29]. According to [30], the adoption of agroforestry is positively related to adequate knowledge, a positive attitude, and perceived low implementation constraints.

Research simultaneously focusing on intrinsic and extrinsic factors’ role in the up-take of agroforestry practices is limited to date. Some studies known to the authors that have attempted to simultaneously assess the effect of extrinsic and intrinsic variables on agroforestry practices adoption include [29,31]. However, these studies did not focus on all three intrinsic variables, and a broader picture is only discovered when they are put together. Therefore, this study aims to add to the literature by empirically examining the role of knowledge, attitudes, perceptions, and extrinsic factors in the uptake of agroforestry practices among rural households to better understand the adoption process. Understanding the role of rural communities’ knowledge and how they perceive agroforestry practices is essential since it is recognized as a significant response to the threat of climate change. Given this motivation, the research question is: what is the nature and the extent of the relationship between socio-psychological factors (knowledge, attitudes, and perceptions) and the adoption of agroforestry practices among rural households? The study hypothesized a positive relationship between the socio-psychological factors and the adoption level of agroforestry practices in the study area.

The remainder of this paper is structured into five sections. The following section presents the theoretical framework. This is followed by the research methodology, results, and discussion sections. The final section presents the conclusions and recommendations based on the empirical results.

## 2 Theoretical Framework

**Figure 1:**
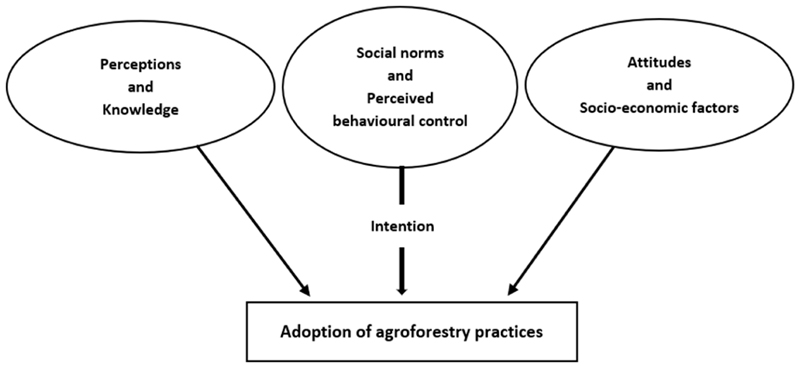
Theoretical research framework (Source: [29,32,34]).

Figure 1 shows a modified theory of planned behaviour (TPB) framework. The TPB is a theoretical framework widely employed to describe and predict an individual’s behaviour. Conscious decision-making and goal-oriented behaviour of an individual are the main focus aspects of TPB [32]. Various studies have applied the theory to explain factors affecting the adoption of agroforestry practices [28,29,32]. It states that the intention to adopt the practices is influenced by attitudes, social norms, and perceived behavioural control [33].

According to [29], the literature on agroforestry practices confirms the adoption of agroforestry (behavioural intention) is significantly and positively associated with the acceptance of support from family, relatives, cooperative members, and friends (social norms), having a positive opinion (attitude), and believing in having the capability to successfully engage in these practices (perceived behavioural control). In the context of agroforestry, behavioural control is associated with the beliefs about the existence of factors that may enhance (e.g., skills and opportunities) or hinder (e.g., financial constraints and inadequate farming assets) the household’s ability to adopt the practice [34].

Following [29,34], the framework also incorporates knowledge, perceptions, and socio-economic characteristics to explain the adoption of agroforestry practices. According to [34], knowledgeable individuals are more confident in adopting new technologies. Individuals’ adoption decisions are also influenced by their perceptions of the advantages and disadvantages of agroforestry practices. Moreover, understanding the agroforestry adoption process also requires an analysis of socio-economic factors [29].

## 3 Materials and Methods

### 3.1 Study Area Description

**Figure 2:**
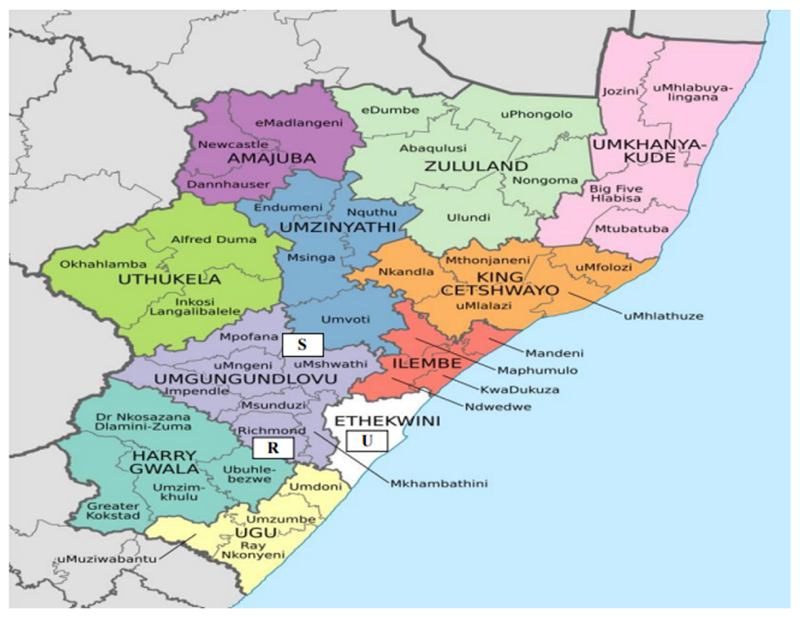
Map showing the KwaZulu-Natal province (Source: [17]). Note: R, S, and U represent Richmond, Swayimane, and Umbumbulu, respectively.

The study was conducted in Swayimane, Umbumbulu, and Richmond, located in KwaZulu-Natal (KZN) province. Figure 2 shows the research study area. Swayimane is located in uMshwathi Local Municipality under the Gcumisa Traditional Authority. The area comprises good precipitation (500 to 800 mm yr^−1^), fertile soils, and a population of 6856 [17]. Umbumbulu is in ward 100 under eThekwini Metropolitan Municipality. The area is characterized by small-scale subsistence farming. Richmond is located in Richmond Local Municipality and comprises seven wards. The population of the area is approximately 65,793 [35].

The province was selected for the study due to its immense potential for agroforestry practices. Its agricultural sector contributes significantly to the national gross domestic product and provides a major source of employment for many rural households [36]. However, extreme changes in rainfall patterns and increases in temperatures negatively affect crop productivity [6]. This condition calls for a significant transformation in the province’s agricultural sector to ensure adequate food supplies and improved food and nutrition security among rural households in South Africa.

The choice of the three study sites was based on the presence of agricultural land uses, which integrate trees and shrubs with agricultural crops and/or livestock, or both. Most of the households are involved in homestead agroforestry practices, demonstrating an immense potential for sustainable agricultural activities. The commonly grown crops include maize, cabbage, sweet potatoes, cassava, and beans. Fruit trees such as bananas, oranges, peaches, avocados, and guava are also grown. The common livestock owned by households are domestic chickens, cattle, and goats. Stokvel clubs, churches, community meetings, and social media applications such as WhatsApp and Facebook are used as platforms for sharing knowledge, experiences and learning, and for accessing support services. Moreover, the study sites have limited economic and job opportunities.

### 3.2 Sampling Method

Both purposive and stratified random sampling methods were applied to select the respondents. The study purposively selected municipalities with households involved in some form of farming. For the purpose of stratifying, the households were classified into three groups, namely, *agrisilvicultural, silvopastoral*, and *agrosilvopastoral*. The sampling approach for the study was driven by two deliberations. Firstly, the existence of different types of agroforestry practices adopted by rural households in the KZN province. Secondly, to align the study with the government’s goal of promoting the farming sector as a key contributor to job creation and rural development. Prior to the household survey, focus group discussions were held. The quantitative survey randomly selected and interviewed a sample of 317 households. However, twelve incomplete questionnaires were discarded. This yielded a total of 305 questionnaires valid for analysis: Swayimane (92), Umbumbulu (103), and Richmond (110). According to [37], a sample size greater than or equal to 50 is considered reasonably large and adequate to conduct significant statistics.

### 3.3 Data Collection

The survey was conducted between September and October 2022 by trained enumerators. The data collection instrument and procedures were approved by the Humanities and Social Sciences Research Ethics Committee (HSSREC) of the University of KwaZulu-Natal (protocol reference number: HSSREC/00003793/2022). A structured and pre-tested questionnaire was utilized to collect data. The questionnaire encompassed questions about socio-economic and demographic factors (e.g., age, household size, gender, farming experience, education level, access to agricultural extension services, etc.), livelihood assets, and the household’s involvement in agroforestry practices. The household’s physical assets were used as indicators of their resource availability and status of wealth [38]. Following previous studies [39,40] the questionnaire also included five-point Likert scale statements to measure respondents’ knowledge, attitudes, and perceptions toward agroforestry practices. Appendix A shows a brief summary of all explanatory variables (socio-economic and demographic, knowledge, perceptions, and attitudes of respondents), the expected results, and related literature. More-over, Appendix B shows questions related to the agroforestry practice status of sampled households and related literature.

The questionnaire was pre-tested for two reasons: to inspect the validity, cultural sensitivity, flow, and questions’ consistency, and to facilitate and refine the translation of questions to the local language. For consistency motives, a similar questionnaire was utilized for all the respondents. Moreover, semi-structured interviews with open-ended questions were administered via focus group discussions to complement information captured through the use of a questionnaire. All the interviews were conducted in-person to control respondents’ unfamiliarity to complete the questionnaire and lessen non-response error.

### 3.4 Statistical Data Analysis

The survey data were analyzed using the International Business Machines (IBM) Statistical Package for Social Sciences (SPSS) version 28 and STATA SE version 17. The descriptive statistics of socio-economic and demographic characteristics of households were reported in terms of percentages, means, and standard deviations (SD). Statistical analysis by means of principal component analysis and a binary logistic regression model was also conducted to determine factors linked with the adoption of agroforestry practices by rural households.

#### 3.4.1 Principal Component Analysis

The principal component analysis (PCA) was used to create indices for the independent variables designed to represent rural households’ knowledge, perceptions, and attitudes toward agroforestry practices. PCA is a widely used multivariate data analysis technique that linearly transforms an original set of variables into a new set of uncorrelated and orthogonal variables called principal components (PCs) [37,41]. The objective is to reduce the number of variables to a few factors without losing most of the original information. The PCs can be related to the original variables as:

(1) $$\begin{array}{l} PC_{1}=a_{11}\chi_{1}+a_{12}\chi_{2}+\ldots +a_{1n}\chi_{n}\\ PC_{m}=a_{m1}\chi_{1}+a_{m2}\chi_{2}+\ldots +a_{mn}\chi_{n} \end{array}$$

where *a*_*mn*_ denotes the weight for the *m*^*th*^ PC and the *n*^*th*^ variable, *χ*_*n*_ denotes the *n*^*th*^ variable. Bartlett’s test of Sphericity was applied to check if the observed correlation matrix diverges significantly from the identified matrix. A statistically significant value (*p* < 0.10) meant that there was sufficient correlation, and the data were appropriate for PCA. Moreover, the Kaiser–Mayer–Olkin (KMO) measure of sampling adequacy was also applied, with a value > 0.5 implying PCA could be performed. The Kaiser criterion which recommends retaining factors with eigenvalues > 1 was used for the factor retention decision. The varimax rotation method was used to enhance the interpretability of the PCA results. Factor loadings greater than 0.50 were considered to have a strong influence on the PCs and were interpreted.

#### 3.4.2 Binary Logistic Regression Model

The econometric models that are usually utilized to examine the adoption of innovative systems comprise logistic regression (logit and probit) and linear regression models. The regression models’ response variable is a linear function and follows a normal distribution [42]. Logistic regression models are non-linear and have a binary response variable. In this study, the response variable is binary (i.e., 1 for adoption and 0 for non-adoption). There-fore, following several studies [29,43–45], a binary logistic regression model was utilized to examine the determinants of agroforestry adoption among rural households. This model is a maximum likelihood estimation technique used to calculate the relationship between a binary dependent variable and a set of independent variables. It estimates the likelihood that a feature is present, or otherwise. That is, the probability of adopting agroforestry practices is specified by *P*_*i*_, while that of not adopting is specified by 1 *− P*_*i*_. The odds ratio is expressed as *P*_*i*_/(1 *−P*_*i*_). The log of the odds ratio which is projected by the logit technique is derived from the natural logarithm of the odds ratio [44,46].

The model is more realistic, robust to outliers, and assumes a logistic distribution of errors, contrary to the probit model which is sensitive to outliers and assumes normally distributed errors [29,47,48]. Moreover, the logit model has two practical advantages, namely, simplicity and interpretability. Its inverse linear transformation can be construed directly as a logarithm of likelihoods, while the probit’s inverse transformation does not have a direct interpretation [49]. It also incorporates the natural logarithm of an odds ratio to overcome difficulties of the ordinary least squares (OLS) regression in treating binary outcomes [50]. Unlike OLS, the logistic regression model accommodates a non-linear relationship between the response and explanatory variables. For more details on the features of the logistic regression model see [37] (pp. 553–555). In this study, a binary logistic regression model characterizing the adoption of agroforestry practices is denoted as follows:

(2) $$\text{ln}\left[P_{i}/\left(1-P_{i}\right)\right]=\beta_{0}+\beta_{1}\chi_{1i}+\beta_{2}\chi_{2i}+\ldots +\beta_{n}\chi_{ni}+\varepsilon_{i}$$

where ln[*P*_*i*_/(1 *−P*_*i*_)] denotes log odds ratio, *P* is the probability of the outcome (i.e., 1 if the household practices agroforestry and 0 otherwise), *i* is observation in the *i*^*th*^ sample, *β*_0_ is the constant, *β*_1_, *β*_2_,… *β*_*n*_ are coefficients of independent variables *χ*_1_, *χ*_2_, … *χ*_*n*_, and *ε* is the normally distributed error term. The coefficients of independent variables and the odds ratio of the regression model were used to interpret the relationship between the independent and explanatory variables. Marginal effects were also calculated to show how a dependent variable (outcome) changes if a specific explanatory variable varies.

The Hosmer–Lemeshow and likelihood ratio tests were utilized to evaluate the goodness of fit of the model. A statistically insignificant Hosmer–Lemeshow test value (*p*-value > 0.05) indicates a good fit of the model [51]. In contrast, a statistically significant likelihood ratio chi-square (Chi^2^) test value (*p*-value < 0.05) supports the existence of a relationship between the dependent variable and independent variables. The Wald test was used to test the significance of individual logistic regression coefficients for each variable. A classification table showing the percentage of all cases correctly predicted was also used to assess the model’s overall accuracy [52,53]. Moreover, the variance inflation factor (VIF) was calculated to check for multicollinearity in the outcome equation. The average VIF below the critical value of 10 indicates the absence of multicollinearity [37].

## 4 Results

### 4.1 Socio-Economic and Demographic Characteristics of Sampled Households

Descriptive statistics for socio-economic and demographic variables are presented in Table 1. The average age of sampled household heads and farming experience were 61.83 and 19.99 years, respectively. Sampled household heads had low levels of education. This is consistent with the literature, which indicated that most household heads in KZN attained a primary level of education. The estimated mean of the log of physical assets’ total value (e.g., radio, television, tractor, and water tank) was 9.49. Sampled households had access to small land sizes with an average of 1.33 hectares and owned livestock such as cattle, goats, and domestic chickens. About 92.5% of household heads were members of different social groups. Moreover, the results showed that access to agricultural extension was low (17.4%). Most rural households complained about inadequate and ineffective extension services. The results also showed that 42% of households were male-headed.

### 4.2 Agroforestry Practices Involvement and Willingness to Expand and Adopt

Table 2 shows the agroforestry practices status of sampled rural households. Umbumbulu had a higher number of households involved in agroforestry practices (95.1%), followed by Richmond (89.1%) and then Swayimane (85.9%). A dominant agroforestry type was the combination of trees/shrubs with agricultural crops and livestock (79.6%).

The results showed that 88% of interviewed households were willing to expand their practices if an opportunity arose. For example, they were willing to have more livestock and plant more trees to increase the size of their agroforestry practices. However, land scarcity, access to agricultural inputs and equipment, financial constraints, and water availability were barriers to their ability to expand. Moreover, 83.3% of households in Richmond were willing to adopt agroforestry. Inadequate knowledge about tree planting and respondents’ age were among the factors contributing to unwillingness to adopt agroforestry practices.

### 4.3 Principal Component Analysis (PCA) Results

The PCA-derived agroforestry practices knowledge indices are presented in Table 3. Three PCs accounting for 65.952% of the total variation in the data were retained. The first component (PC_K1_) was closely related to knowing agroforestry practices. The second component (PC_K2_) was found to be closely associated with maximized land usage. This is in line with previous studies which indicated that agroforestry maximizes land usage. The third component (PC_K3_) was closely related to agroforestry being against animal grazing. Some respondents indicated that planting trees on farmland reduces grazing land for livestock.

The PCA-derived agroforestry practices perception indices are presented in Table 4. Four PCs accounting for 55.49% of the total variation in the data were retained. The first component (PC_P1_) represents households who perceive agroforestry as expensive and labour-intensive. Rural households often lack the ability to attain optimal levels of financial capital which hinders their potential to uptake agroforestry practices. Achieving the long-term benefits of agroforestry requires high initial investment which could be expensive for these households. Moreover, agroforestry practices may have high labour requirements such as digging a hole to plant a tree or shrub. The second component (PC_P2_) was closely related to hindering the use of modern farm implements. The third component (PC_P3_) was found to be closely related to profitability. Agroforestry systems are more profitable because they create various income streams through tree products, crops, and livestock sales. The fourth component (PC_P4_) was found to be closely related to the technicality of agroforestry systems. Most sampled households indicated that agroforestry practice is not properly understood due to its technicality. This indicates a lack of information and skills which may be due to a lack of access to agroforestry extension officers.

The PCA-derived agroforestry practices attitudes indices are presented in Table 5. This measures the intention levels of households to plant trees on their farmland. Three PCs accounting for 52.73% of the total variation in the data were retained. The first component (PC_A1_) showed that households viewed agroforestry as a worthwhile investment. It was found to be closely related to fuel and furniture wood provision. Most respondents considered planting trees vital because they provide fuelwood as a source of energy for heating, boiling water, and cooking. The second component (PC_A2_) was found to be closely related to reduced crop yields. This may result from the presence of trees on a limited amount of farmland which interferes with crop production. For example, some sampled rural households indicated that tree shading reduced crop yields. The third component (PC_A3_) was closely related to controlling air pollution. This is in line with the literature which reported that planting trees greatly reduces greenhouse gas emissions and improves atmospheric carbon dioxide capture.

### 4.4 Binary Logistic Regression Model Results

A binary logistic regression model was used to examine the determinants of agro-forestry practices adoption (Table 6). Post-estimation diagnostic tests were conducted to check the model’s goodness of fit. A statistically insignificant Hosmer–Lemeshow test value (*p*-value = 0.667) indicated a good fit of the model. The likelihood ratio chi-square test value indicated that the model was statistically significant and had a strong explanatory power (*p*-value = 0.000). Therefore, this study rejected the null hypothesis that the model without explanatory variables and the model with explanatory variables were similar. According to Cox and Snell R^2^, Nagelkerke R^2^, and McFadden R^2^ values, the dependent variable defines 19%, 40%, and 32.7% of the variance in independent variables, respectively. Moreover, the model correctly classified 91.80% of the cases and had a statistically significant Wald test (*p*-value = 0.000). Multicollinearity was not a challenge since the VIFs had an average of 1.31, well below the threshold.

## 5 Discussion

This study looked at the socio-psychological factors (knowledge, attitudes, and perceptions) and their role in the adoption of agroforestry practices among rural households. The results showed a positive relationship between these three factors and adoption of the practices (Table 6). According to the argument in the introduction of this paper, focusing on all three intrinsic variables is essential to better understand the adoption process for agroforestry practices among rural households. Therefore, the findings of this study confirm that including knowledge, perceptions, and attitudes variables in one analysis improves the understanding of adoption decisions. This demonstrates the novelty of this study that all these variables are crucial. Other countries can benefit from this study by ensuring that they include all intrinsic factors in their future adoption studies and policy formulation.

Household heads who were more knowledgeable about agroforestry practices had a higher adoption capability. This is consistent with studies from Nigeria [31] and Pakistan [29] which emphasized that adequate knowledge promotes adopting agroforestry practices. Marginal effect’s results showed that the likelihood of adopting agroforestry was 1.2% higher among knowledgeable household heads than those without knowledge. Therefore, educating rural households about trees’ economic and environmental benefits could increase tree cover in the agricultural landscape. Sampled households who perceived agroforestry as a profitable practice were more likely to adopt agroforestry practices. This is consistent with [29] who reported a positive relationship between perceptions and agroforestry adoption. According to [54], most rural households perceive agroforestry as a profitable practice compared to a monoculture production system. The results also showed that sampled households who agreed that planting trees provides fuelwood and furniture wood (i.e., positive attitude) had a greater likelihood of adopting the agroforestry practices, *ceteris paribus*. Households using firewood as an energy source tend to plant more trees on their farmland than those using electricity, paraffin, and gas [29,55]. Moreover, tree species such as teak provide the raw material for furniture.

In this study, a distinction between age and farming experience was made because a 50-year-old household head, for instance, might have commenced farming at the age of 40 and another of the similar age might have commenced at the age of 30. Thus, their knowledge, skills, and motives for farming might differ based on their age and experiences [56]. The results indicated a positive and significant relationship between the age of the household head and the adoption of agroforestry practices. That is, a one-year rise in household head’s age would increase the probability of adopting agroforestry by 5.5%, *ceteris paribus*. This is in line with previous studies [23,42] which indicated that older household heads are more likely to adopt agroforestry practices than younger ones. Younger individuals perceive agroforestry practice as a long-term method due to the slow growth rate of tree species compared to cash crops [29]. This calls for a need to promote fast-growing species in farmlands. Adoption of agroforestry practices was also found to be positively associated with the farming experience. This is consistent with [44] who reported that more experienced households have the knowledge and skills to manage their on-farm activities effectively.

The relationship between household heads’ education level and agroforestry practices adoption was positive and significant. Increasing household head’s education by one year increases the probability that they would adopt agroforestry by 12.2%, *ceteris paribus*. This is because education improves access to knowledge, understanding of technologies, and opportunities’ identification. Previous studies such as [45] also asserted that educated individuals tend to have the capacity to adopt agroforestry practices. The results indicated that the estimated coefficient of land size was positively related to the adoption of agroforestry practices, *ceteris paribus*. The odds of adopting agroforestry practices were 5.351 times higher among households with larger land sizes than those with smaller land sizes. Bigger land sizes enable rural households to accommodate trees, shrubs, agricultural crops, and livestock for optimal benefits. This is in line with numerous studies [44,46] that reported a positive relationship between land size and agroforestry adoption. According to [57], agroforestry practices are less likely to be economically feasible on small land sizes. The results regarding the marginal effect indicated that households with bigger land sizes were 3.8% more likely to adopt agroforestry practices.

## 6 Conclusions

This study empirically investigated the determinants of agroforestry practices adoption among rural households. It emphasized the importance of incorporating socio-psychological factors, such as households’ perceptions and attitudes, to effectively design and implement agroforestry projects. Knowledge, attitudes, and perceptions towards agroforestry were found to impact the adoption of the practice positively. These findings support the hypothesis that socio-psychological factors have a positive impact on the adoption of agroforestry practices among rural households. The results also showed that agroforestry adoption was significantly affected by age, farming experience, education level, and land size. Therefore, the study concludes that socio-economic and socio-psychological factors are associated with agroforestry adoption. Considering rural households’ socio-economic characteristics, knowledge, attitudes, and perceptions when designing agroforestry projects is recommended.

Extension officers, climate change champions, researchers, policymakers, and other stakeholders need to join forces in public–private partnerships to collectively participate in distributing adequate knowledge on agroforestry practices and their advantages to rural households. The use of media to raise awareness and information on the impact of changing climate and the benefits of agroforestry in locally understood languages and the implementation of training programs with practical demonstration is essential in promoting the level of adoption and encouraging rural households to protect on-farm trees and shrubs. Moreover, addressing institutional and service constraints such as land scarcity, access to tree samplings and agricultural equipment, financial constraints, water availability, and inadequate knowledge is vital to enhance the adoption and expansion of agroforestry practices. Actively involving rural households in the design of agroforestry programs is also important to develop programs that meet households’ needs and preferences, improve food and nutrition security, and address climate change related risks within rural communities.

Moreover, as mentioned earlier, one possible reason for the lack of research on understanding the relationship between intrinsic factors and agroforestry adoption may be methodological challenges associated with measuring individual’s perceptions of farming systems. This study then used five-point Likert scale statements to measure respondents’ knowledge, attitudes, and perceptions. Using numerous statements to measure each factor, rather than one statement generated a wealth of knowledge from the analysis. The results showed that certain aspects of knowledge, perceptions, and attitudes were significant and critical to rural households’ agroforestry adoption decisions, while others were not. For example, agroforestry knowledge, perceptions of profitability, and positive attitudes affected adoption decisions. Therefore, it is recommended that other studies use a similar approach to measure intrinsic variables for better results.

## Supplementary Material

Appendix

## Figures and Tables

**Table 1:** Socio-economic factors, their means, standard deviations, and percentages.

Variable	Description	Mean	SD	%
**Continuous variables**				
Age	Household head age (Years)	61.83	14.05	-
Experience	Household head farming experience (Years)	19.99	15.36	-
Education	Household head education level (Years of schooling)	5.48	4.90	-
Land size	Land size household has access to (Hectares)	1.33	1.22	-
Total livestock units	Tropical livestock units	1.75	3.05	-
Assets	Log of the total value of physical assets	9.49	1.33	-
Off-farm income	Log of the annual income from non-farm activities	10.88	0.86	-
**Dummy variables**				
Group membership	Membership in groups (1 = Yes; 0 = otherwise)	-	-	92.5
Extension	Agricultural extension (1 = Yes; 0 = otherwise)	-	-	17.4
Gender	Household head gender (1 = Male; 0 = otherwise)	-	-	42.0

Source: Authors’ own analysis.

**Table 2:** Agroforestry practices status of sampled households.

	Swayimane	Umbumbulu	Richmond	Total
Households involved in agroforestry (%)	85.9	95.1	89.1	90.2
Agroforestry-type households involved in *Trees/shrubs and agricultural crops (%)*	34.2	10.2	5.1	15.3
*Tress/shrubs and livestock (%)*	3.8	1.0	10.2	5.1
*Trees/shrubs with agricultural crops and livestock (%)*	62.0	88.8	84.7	79.6
Households willing to expand agroforestry (%)	91.1	88.8	84.7	88.0
Households willing to adopt agroforestry (%)	69.2	100.0	83.3	80.0

Source: Authors’ own analysis.

**Table 3:** Households’ knowledge of agroforestry practices.

Variables	Principal Components		
	PC_K1_—Agroforestry Knowledge	PC_K2_—Land Utilisation	PC_K3_—against Animal Grazing
Before this interview, I knew about forestry farming	0.636	0.411	0.014
I have always known about agroforestry practices although I did not know the exact wording	0.751	−0.115	−0.159
I have always known and understood what agroforestry practices are	0.760	0.003	0.230
Agroforestry is against the practice of animal grazing	0.030	0.036	0.946
Agroforestry maximizes land usage	0.011	0.818	−0.150
Agroforestry guarantees consistent supply to the markets	0.046	0.695	0.220
Eigenvalue	1.74	1.18	1.01
% of variance	28.91	19.71	16.91
Cumulative % of variance	28.91	48.62	65.52

Note: Only component loadings greater than |0.50| are included in the interpretation; KMO = 0.60 and Bartlett’s Test of Sphericity Chi^2^ = 121.93, *p*-value = 0.000 (Source: Authors’ own analysis).

**Table 4:** Households’ perceptions towards agroforestry practices.

Variables	Principal Components			
*Agroforestry Practice Is*:	PC_P1_—Expensive and Labour-Intensive	PC_P2_—Incompatibility to Modern Farm Equipment	PC_P3_—Profitable	PC_P4_—Technical
Difficult to practice	0.613	0.190	−0.031	0.233
A common practice in this area	−0.088	0.335	0.637	−0.214
Can increase farm productivity	0.003	−0.124	0.651	0.277
Not properly understood because of its technicality	−0.040	0.144	−0.018	0.829
Time consuming	0.673	−0.036	0.119	0.086
Not profitable	−0.036	0.141	−0.670	0.004
Expensive to practice	0.750	0.078	−0.153	0.141
Labour-intensive	0.804	0.057	0.013	−0.135
Cannot be practiced on small piece of land	0.278	0.669	−0.002	0.246
Hinders the use of modern farm implements	−0.004	0.829	−0.093	−0.010
Not meant for low-income/smallholder farmers	0.221	0.009	0.076	0.528
Eigenvalue	2.45	1.35	1.26	1.04
% of variance	22.26	12.28	11.48	09.47
Cumulative % of variance	22.26	34.55	46.02	55.49

Note: Only component loadings greater than |0.50| are included in the interpretation; KMO = 0.66 and Bartlett’s Test of Sphericity Chi^2^ = 389.27, *p*-value = 0.000 (Source: Authors’ own analysis).

**Table 5:** Households’ attitudes towards agroforestry practices.

Variables	Principal Components		
*Planting Trees on My Land Will:*	PC_A1_—Positive Attitudes	PC_A2_—Negative Attitudes	PC_A3_—Environmental Contribution
Increase household income	0.600	−0.241	0.247
Provide fuelwood and furniture wood	0.719	0.030	0.093
Control soil erosion	0.203	−0.082	0.797
Control air pollution	0.062	0.182	0.837
Cause hindrance in agricultural operations	−0.042	0.631	−0.060
Cause shade that will reduce the yield of crops	0.126	0.722	0.245
Incur more cost	0.193	0.678	−0.212
Provide harbor to insects, pests, and diseases	−0.206	0.581	0.182
Provide shade for human beings and animals	0.671	0.050	0.188
Be a long-time land utilization	0.618	0.094	−0.106
Eigenvalue	2.22	1.81	1.25
% of variance	22.18	18.07	12.48
Cumulative % of variance	22.18	40.25	52.73

Note: Only component loadings greater than |0.50| are included in the interpretation; KMO = 0.64 and Bartlett’s Test of Sphericity Chi^2^ = 408.83, *p*-value = 0.000 (Source: Authors’ own analysis).

**Table 6:** Determinants of agroforestry practices adoption: Binary logistic regression model results.

Variables	Coef.	Sig.	Sth. Err.	Wald	Odds Ratio	Marginal Effect
Age	0.055 **	0.046	0.028	3.984	1.057	0.001
Experience	0.038 *	0.080	0.021	3.057	1.038	0.001
Education	0.122 *	0.076	0.069	3.150	1.130	0.003
Extension	−0.779	0.325	0.790	0.971	0.459	−0.023
Gender	−0.272	0.599	0.517	0.277	0.762	−0.006
Land size	1.677 ***	0.000	0.437	14.744	5.351	0.038
Total livestock units	−0.016	0.866	0.093	0.028	0.984	−0.000
Assets	−0.269	0.207	0.214	1.592	0.764	−0.006
Group membership	−1.517	0.153	1.062	2.038	0.219	−0.020
Off-farm income	0.456	0.178	0.339	1.811	1.577	0.010
Agroforestry knowledge (PC_K1_)	0.548 **	0.039	0.266	4.251	1.730	0.012
Land utilisation (PC_K2_)	−0.425	0.159	0.301	1.988	0.654	−0.010
Against animal grazing (PC_K3_)	0.369	0.211	0.295	1.563	1.447	0.008
Expensive and labour-intensive (PC_P1_)	−0.020	0.949	0.307	0.004	0.980	−0.000
Incompatibility to modern farm equipment (PC_P2_)	0.134	0.650	0.295	0.206	1.143	0.003
Profitable (PC_P3_)	0.934 ***	0.002	0.306	9.333	2.544	0.021
Technical (PC_P4_)	−0.452	0.140	0.307	2.173	0.636	−0.010
Positive attitudes (PC_A1_)	0.633 **	0.021	0.275	5.311	1.883	0.014
Negative attitudes (PC_A2_)	0.409	0.179	0.305	1.804	1.506	0.010
Environmental contribution (PC_A3_)	−0.013	0.957	0.246	0.003	0.987	−0.000
Constant	−4.045	0.212	3.242	1.556	0.018	
Multicollinearity test	1.31					
Number of cases correctly classified	91.80%					

Note: ***, ** and * indicate the level of significance at 1%, 5% and 10%, respectively; Hosmer–Lemeshow test Chi^2^ = 5.825, *p*-value = 0.667; –2 Log likelihood = 131.935; Likelihood ratio Chi^2^ = 64.159, *p*-value = 0.000; Wald test = 132.778, *p*-value = 0.000; Cox and Snell R^2^ = 0.190; Nagelkerke R^2^ = 0.400; McFadden R^2^ = 0.327 (Source: Authors’ own analysis).

## Data Availability

The data presented in this study are available on request from the corresponding authors. The data are not publicly available due to confidentiality.
