# Supplementary material for: Factors Influencing the Uptake of Agroforestry Practices among Rural Households: Empirical Evidence from the KwaZulu-Natal Province, South Africa
Source: Forests. Author manuscript; Available in PMC 2024 Dec 9. (PMC7617150; doi:10.3390/f14102056)
Supplement: Appendix [file EMS197322-supplement-Appendix.pdf]

## Appendix A

**Table A1.** Explanatory variables (socio-economic and demographic, knowledge, perceptions, and attitudes of respondents), the expected results, and related literature.

| Variable | Expected Outcome | References |
|---|---|---|
| Socio-economic and demographic | | |
| Age | Positive | [23,42] |
| Experience | Positive | [44] |
| Education | Positive | [45,58] |
| Extension | Positive | [44,59] |
| Gender | Positive | [42] |
| Land size | Positive | [29,60] |
| Total livestock units | Positive | [61] |
| Assets | Positive | [62] |
| Group membership | Positive | [44] |
| Off-farm income | Positive | [63] |
| Knowledge of agroforestry practices * | | |
| a. Before this interview, I knew about forestry farming | Positive | [31] |
| b. Before this interview, I did not know I can combine trees, crops, and livestock businesses | Negative | [31] |
| c. I have always known about agroforestry innovations although I did not know the exact wording | Positive | [31] |
| d. I have always known and understood what agroforestry innovations are | Positive | [31] |
| e. Agroforestry is against the practice of animal grazing | Negative | [31] |
| f. Agroforestry maximizes land usage | Positive | [31] |
| g. Agroforestry guarantees consistent supply to the markets | Positive | [40] |
| Perceptions towards agroforestry practices * | | |
| a. Agroforestry is difficult to practice | Negative | [23,54] |
| b. Agroforestry is a common practice in this area | Positive | [23] |
| c. Agroforestry practice can increase farm productivity | Positive | [23,54] |
| d. Agroforestry practice is not properly understood because of its technicality | Negative | [23] |
| e. Agroforestry practice is time consuming | Negative | [23] |
| f. Agroforestry practice is not profitable | Negative | [23] |
| g. Agroforestry is expensive to practice | Negative | [23] |
| h. Agroforestry practice is labour-intensive | Negative | [23] |
| i. Agroforestry practice cannot be practiced on small piece of land | Positive/negative | [23] |
| j. Agroforestry practice hinders the use of modern farm implement | Negative | [23] |
| k. Agroforestry practice is not meant for low-income/smallholder farmers | Negative | [23] |
| Attitudes towards agroforestry practices: "Planting trees on my agricultural land will..." * | | |
| a. Increase household income | Positive | [39,64] |
| b. Provide fuel wood and furniture wood | Positive | [39,64,65] |
| c. Control soil erosion | Positive | [39,64] |
| d. Control air pollution | Positive | [39] |
| e. Improve soil conservation | Positive | [29] |
| f. Cause hindrance in agricultural operations | Negative | [39,64] |
| g. Cause shade that will reduce the yield of crops | Negative | [39,64] |
| h. Incur more cost | Negative | [39] |
| i. Provide harbor to insects, pests and diseases | Negative | [39] |
| j. Provide shade for human beings and animals | Positive | [39,66] |
| k. Be a long-time land utilization | Positive | [39] |

Note: * 1 = Strongly disagree; 2 = Disagree; 3 = Do not know (neutral); 4 = Agree; 5 = Strongly agree.

## Appendix B

**Table A2.** Agroforestry practices status of sampled households.

| Question | References |
|---|---|
| 1. Are you involved in agroforestry practice? *1 = Yes 0 = No* | [23,31] |
| 2. If Yes to 1, which agroforestry type(s) are you involved in? *1 = Trees/shrubs and agricultural crops 2 = Trees/shrubs and livestock 3= Trees/shrubs with agricultural crops and livestock* | [26] |
| 3. If Yes to 1, would you be interested in expanding your agroforestry practice if an opportunity arises? *1 = Yes 0 = No* | Authors |
| 4. If Yes to 3, what are the factors holding you up to expand agroforestry practice? | [67] |
| 5. If No to 1, would you be interested in adopting agroforestry practice if an opportunity arises? *1 = Yes 0 = No* | Authors |
| 6. If No to 5, why? | Authors |
